# The predictive nature of transcript expression levels on protein expression in adult human brain

**DOI:** 10.1186/s12864-017-3674-x

**Published:** 2017-04-24

**Authors:** Amy L. Bauernfeind, Courtney C. Babbitt

**Affiliations:** 1 Department of Neuroscience, Washington University Medical School, St. Louis, MO 63110 USA; 2 Department of Anthropology, Washington University in St. Louis, St. Louis, MO 63130 USA; 3 Department of Biology, University of Massachusetts Amherst, Amherst, MA 01003 USA

**Keywords:** RNA-Seq, Gene, Proteomics, Chimpanzee

## Abstract

**Background:**

Next generation sequencing methods are the gold standard for evaluating expression of the transcriptome. When determining the biological implications of such studies, the assumption is often made that transcript expression levels correspond to protein levels in a meaningful way. However, the strength of the overall correlation between transcript and protein expression is inconsistent, particularly in brain samples.

**Results:**

Following high-throughput transcriptomic (RNA-Seq) and proteomic (liquid chromatography coupled with tandem mass spectrometry) analyses of adult human brain samples, we compared the correlation in the expression of transcripts and proteins that support various biological processes, molecular functions, and that are located in different areas of the cell. Although most categories of transcripts have extremely weak predictive value for the expression of their associated proteins (R^2^ values of < 10%), transcripts coding for protein kinases and membrane-associated proteins, including those that are part of receptors or ion transporters, are among those that are most predictive of downstream protein expression levels.

**Conclusions:**

The predictive value of transcript expression for corresponding proteins is variable in human brain samples, reflecting the complex regulation of protein expression. However, we found that transcriptomic analyses are appropriate for assessing the expression levels of certain classes of proteins, including those that modify proteins, such as kinases and phosphatases, regulate metabolic and synaptic activity, or are associated with a cellular membrane. These findings can be used to guide the interpretation of gene expression results from primate brain samples.

**Electronic supplementary material:**

The online version of this article (doi:10.1186/s12864-017-3674-x) contains supplementary material, which is available to authorized users.

## Background

Next generation sequencing, including RNA-Seq, allows researchers to investigate transcript expression using label-free technology, and its relative ease of use has made this method the dominant technology for assessing molecular phenotype. When interpreting transcriptomic results, the assumption is frequently made that the expression level of a transcript reflects that of the downstream protein, suggesting the equivalence of these two molecules. However, the relationship between these two aspects of molecular phenotype has yet to be fully understood. In fact, the correlation between transcript expression levels and their protein products have generally been found to be quite low and may vary across tissues and cell types [1–5], calling into question what biological significance can be drawn from transcriptomic and proteomic results.

Our earlier study [6] explored transcript (RNA-Seq) and protein (liquid chromatography with tandem mass spectrometry [LC/MS/MS]), expression in the anterior cingulate cortex (ACC) and caudate nucleus (CN) of humans and chimpanzees in order to determine if differential expression analyses of these two molecules resulted in different interspecific biological signals. Importantly, we reported that both species display a lower degree of correlation between transcript and protein expression levels (human R^2^ = 0.03; chimpanzee R^2^ = 0.04) than typically reported in other organisms and tissues [1, 2]. The correlation between transcript and protein expression in mammalian cells is generally modest, ranging from 9% in human monocytes to 40% in mouse fibroblasts [7, 8], but these and other inquiries into the relationship between transcript and protein expression levels have done so using homogenous cell cultures in an effort to limit confounding variables [9]. Many transcriptomic studies, however, must overcome additional challenges imposed by longer postmortem intervals (PMIs) and greater cellular heterogeneity than these carefully controlled studies.

In the spirit of exploring the correlation between transcript and protein expression that should be expected from non-model samples, our objective was to determine how transcript expression predicts protein expression within molecular categories that are specific to brain tissue (eg. ‘synapse’) and with limitations that are common in many studies of molecular expression (ie. PMIs of up to 8 h and heterogenous cell populations). We predict that molecules within the same Gene Ontology (GO) attributes (i.e. biological process, molecular function, or cellular component) may share regulatory mechanisms associated with synthesis and degradation, which may affect the degree of correlation between transcript and protein expression levels [10, 11]. Here, we ask whether there are certain classes of transcripts that are more predictive of protein expression levels than others by using transcriptomic and proteomic expression from our previously published dataset [6]. Specifically, we examined expression levels of transcripts and proteins by GO category to determine if different predictive relationships (coefficients of determination, R^2^ values) exist between molecules that participate in certain functions or are located in certain parts of the cell. Are there molecular attributes that suggest a stronger predictive relationship between transcripts and proteins than others? Strong predictive relationships suggest that the results of RNA-Seq would be informative of expression levels of downstream proteins, while the expression levels of classes of transcripts with weaker predictive relationships offer little value in predicting downstream protein abundance.

In general, we found that most transcripts have fairly low predictive value for determining protein expression levels, falling within one standard deviation of the mean for randomly associated transcript/protein pairs. However, we found that transcripts that coded for membrane-bound proteins, in particular those that have oxidoreductase and synaptic functions, and protein kinases and phosphatases were most predictive of protein expression. Our results indicate that the predictive value of transcripts is not uniform across all functions or cellular locations, and we explore possible causes of this variation by investigating correlations between categorical R^2^ value and category size, molecular abundance, gene length, and previously published rates of molecular synthesis and degradation [8]. Understanding implicit biases in transcriptomic and proteomic data is fundamental to answering questions pertaining to the molecular phenotype of the brain or any other biological tissue.

## Results

Overall, we report R^2^ values of 0.07 in the expression levels of 815 transcript/protein pairs in the human ACC. This result is higher than we had reported previously [6] because of the inclusion of more transcript/protein pairs in this expanded dataset that is not limited to homologous proteins between humans and chimpanzees. Notably, the coefficient of determination is similar to that reported by Wei and colleagues in adult human and Rhesus macaque brain tissue [12]. We performed ordinary least squares (OLS) regressions on the transcript and protein expression levels within each GO category of biological process, molecular function, and cellular component. In human ACC, 306 categories of biological processes, 125 categories of molecular functions, and 104 categories of cellular components were represented by 10 or more transcript/protein pairs. A complete ordered list of the transcript and protein expression levels in human ACC and the OLS regression results including the predictive natures (R^2^) of GO categories can be found in Additional file 1. When appropriate, transcripts are associated with more than one category, which accounts for the similarities between functional groupings (eg. “organelle outer membrane” and “outer membrane”).

**Fig. 1:**
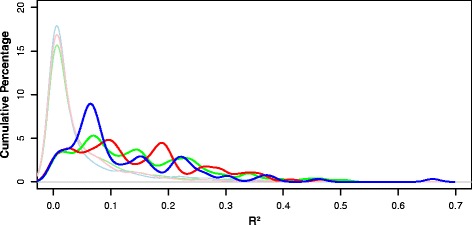
Cumulative percentages of R^2^ values. GO categories of biological process (*green*), molecular function (*red*) and cellular component (*blue*) are plotted together. The lighter green, red, and blue lines represent the results of permutation tests that produced 1000 OLS regressions from randomly sampled transcripts and proteins of equivalent categorical sizes of biological process, molecular function, and cellular component, respectively. The observed data from all three GO annotations have distributions with a positive skew, displaying how there are categories of both molecular function and cellular components that are more predictive of protein expression than randomly associated transcripts and proteins. The data is plotted with a bin width is 0.05, and the representative line is Gaussian smoothed. Duplicate categories that contain the same molecules and have the same R^2^ value as another category are not represented

Descriptive statistics were performed to summarize the R^2^ values across GO categories that contained a minimum of 10 transcript/protein pairs. Because the analyses were dependent upon the categories included and some transcript/protein pairs were represented in more than one category, we used the descriptive statistics as a way of describing what R^2^ results may be expected from specific GO categories, while acknowledging these limitations. Categories that contained the same molecules and had identical R^2^ values as another category, including 45 categories of biological process, 10 categories of molecular function, and 10 categories of cellular component, were deleted from the dataset. Categories of biological process yielded R^2^ values between < 0.01 to 0.51 (mean = 0.15 ± 0.11, median = 0.12). For molecular function, categories produced R^2^ values that ranged from < 0.01 to 0.47 (mean = 0.14 ± 0.10, median = 0.11). Categories of cellular component yielded R^2^ values between < 0.01 to 0.66 (mean = 0.12 ± 0.11, median = 0.08). The central tendency of R^2^ values for categories of biological process was statistically higher than that of cellular components (Mann Whitney U = 13,941, *p* < 0.05), but comparisons revealed that those of the other annotations were statistically equivalent. For each GO annotation, the distributions of categories had positive skews (biological process Shapiro-Wilk = 0.92, *p* < 0.001, skewness = 0.98; molecular function Shapiro-Wilk = 0.95, *p* < 0.001, skewness = 0.71; cellular compartment Shapiro-Wilk = 0.83, *p* < 0.001, skewness = 1.87, respectively), suggesting that while most R^2^ values fall near the median for the annotation other categories have much higher R^2^ values. These categories contain transcripts with greater predictive value. Interestingly, cellular component revealed the largest positive skew, a result largely driven by the highly predictive relationship between transcripts and proteins associated with the cellular ‘outer membrane’ (R^2^ = 0.66) (Fig. 1).

**Fig. 2:**
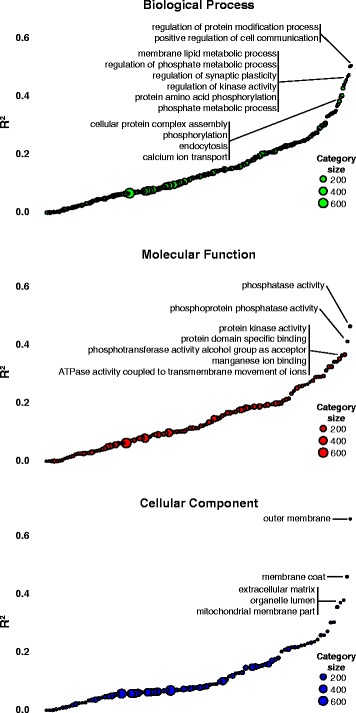
Coefficients of determination for GO categories of molecular function and cellular component. R^2^ values are typically quite low but certain categories (labeled) display greater predictive value than others. These categories have R^2^ values that exceed the mean of the 1000 resampled categories by approximately four standard deviations (two standard deviations above the means for the respective annotations)

We listed the biological processes, molecular functions, and cellular locations of transcripts that were most predictive of the expression levels of their associated proteins (Table 1). Proteins involved in the addition (kinases) or removal (phosphatases) of a phosphate group and transmembrane proteins, including those that are components of receptors or ion channels and have those that have oxidoreductase functions, are among the molecules that display the highest correlation in their expression to their parent transcript (Fig. 2). However, the slopes display a fairly wide range of confidence intervals, reflecting the fact that the relationship between transcript and protein expression can be quite variable within a GO category. The interpretation of our results rely on coefficients of determination, R^2^ values, which are a similar measure of how closely the data are fitted to the regression line, and we interpret this value as a measure of how predictable protein expression levels are from the expression levels of their parent transcripts.

**Table 1:** GO categories with the highest predictive value between transcript/protein pairs

Category	*n*	R^2^	*p*	Slope	Slope CI	Intercept	Intercept CI
**Biological process**							
regulation of protein modification process	10	0.51	0.0211	0.38	0.07 – 0.68	4.06	3.37 – 4.75
positive regulation of cell communication	11	0.50	0.0145	0.47	0.12 – 0.82	3.86	3.05 – 4.68
membrane lipid metabolic process	11	0.47	0.0192	0.75	0.15 – 1.35	2.99	1.52 – 4.46
regulation of phosphate metabolic process	10	0.47	0.0290	0.38	0.05 – 0.7	4.08	3.34 – 4.82
regulation of synaptic plasticity	10	0.46	0.0317	0.73	0.08 – 1.38	3.58	1.88 – 5.29
regulation of kinase activity	15	0.44	0.0070	0.76	0.25 – 1.27	3.16	1.85 – 4.46
protein amino acid phosphorylation	34	0.43	0.0000	0.56	0.33 – 0.79	3.8	3.27 – 4.33
phosphate metabolic process	55	0.40	0.0000	0.57	0.38 – 0.76	3.75	3.31 – 4.19
cellular protein complex assembly	16	0.40	0.0089	0.67	0.2 – 1.15	4.02	2.87 – 5.17
phosphorylation	41	0.38	0.0000	0.56	0.33 – 0.79	3.82	3.3 – 4.35
endocytosis	19	0.37	0.0055	0.57	0.19 – 0.95	4.07	3.2 – 4.93
calcium ion transport	11	0.37	0.0483	0.65	0.01 – 1.3	3.72	2.04 – 5.39
DNA metabolic process	11	0.35	0.0548	0.6	-0.02 – 1.21	3.77	2.48 – 5.05
negative regulation of protein metabolic process	19	0.35	0.0081	0.45	0.13 – 0.76	4.08	3.23 – 4.92
cell cycle	14	0.35	0.0272	0.5	0.07 – 0.93	3.7	2.77 – 4.62
positive regulation of cell proliferation	20	0.34	0.0068	0.41	0.13 – 0.69	4.02	3.50 – 4.54
regulation of hydrolase activity	18	0.34	0.0110	0.53	0.14 – 0.92	3.91	2.98 – 4.84
regulation of actin filament length	12	0.34	0.0477	0.48	0.01 – 0.96	4.18	2.87 – 5.49
cell projection organization	14	0.33	0.0306	0.67	0.07 – 1.26	3.64	2.00 – 5.28
regulation of growth	12	0.33	0.0499	0.51	0 – 1.02	3.93	2.67 – 5.18
regulation of cell growth	10	0.33	0.0824	0.48	-0.08 – 1.04	3.89	2.55 – 5.24
positive regulation of cellular process	64	0.31	0.0000	0.44	0.27 – 0.61	4.16	3.77 – 4.54
protein modification process	65	0.30	0.0000	0.46	0.28 – 0.64	3.96	3.55 – 4.38
**Molecular function**							
phosphatase activity	16	0.47	0.0036	0.97	0.37 – 1.56	2.67	1.24 – 4.11
phosphoprotein phosphatase activity	12	0.41	0.0241	0.72	0.11 – 1.32	3.29	1.83 – 4.74
protein kinase activity	33	0.37	0.0002	0.55	0.28 – 0.81	3.76	3.2 – 4.33
protein domain specific binding	18	0.37	0.0079	0.55	0.16 – 0.93	3.74	2.82 – 4.65
phosphotransferase activity alcohol group as acceptor	45	0.35	0.0000	0.56	0.33 – 0.79	3.8	3.31 – 4.3
manganese ion binding	12	0.34	0.0451	-0.39	-0.76 – - 0.01	5.25	4.52 – 5.98
ATPase activity coupled to transmembrane movement of ions phosphorylative mechanism	17	0.34	0.0135	0.39	0.09 – 0.69	4.74	4.04 – 5.44
GTPase activity	52	0.33	0.0000	0.48	0.29 – 0.68	4.28	3.8 – 4.75
calmodulin binding	31	0.33	0.0008	0.52	0.24 – 0.81	4.11	3.4 – 4.83
ligase activity	20	0.31	0.0102	0.42	0.11 – 0.72	3.85	3.11 – 4.58
**Cellular component**							
outer membrane	10	0.66	0.0043	0.88	0.37 – 1.40	3.4	2.30 – 4.51
membrane coat	13	0.46	0.0107	0.76	0.21 – 1.30	3.58	2.25 – 4.90
extracellular matrix	11	0.38	0.0435	0.67	0.02 – 1.32	3.71	2.16 – 5.25
organelle lumen	10	0.37	0.0612	-0.38	-0.79 – 0.02	5.93	4.98 – 6.89
mitochondrial membrane part	21	0.36	0.0043	0.84	0.30 – 1.39	3.23	1.94 – 4.52
cell surface	12	0.30	0.0630	0.35	-0.02 – 0.72	4.35	3.43 – 5.27
membrane-enclosed lumen	12	0.30	0.0638	-0.37	-0.77 – 0.03	6.01	5.07 – 6.94

Data in bold are the subcategories

### Permutation tests

We performed permutation tests in which random transcripts and proteins were classified into categories to mimic our observed data. The category sizes were sampled and replaced from the actual sizes of our observed categories for molecular function and cellular component to ensure that the range of possible category sizes represented our dataset. The resampling occurred over 1000 iterations. In the permutation test to mimic the annotation of biological process, randomly paired transcripts and proteins yielded R^2^ values that ranged from < 0.01 to 0.60 (mean = 0.05 ± 0.08, median = 0.02). As expected, the central tendency of the observed data was higher than that of the permutation test (Mann Whitney U = 207,103, *p* < 0.001). For molecular function, categories of random transcripts and proteins produced R^2^ values that ranged from < 0.01 to 0.65 (mean = 0.05 ± 0.07, median = 0.02). The R^2^ values for molecular function permutation test compared to the observed data were inequivalent (Mann Whitney U = 94,099, *p* < 0.001) with the median of the observed data greater than that of the permutation test. The permutation test of cellular component categories yielded R^2^ values between < 0.01 and 0.64 (mean = 0.05 ± 0.08, median = 0.01). For cellular component, the central tendency of the observed R^2^ values was higher than that of the permutation test (Mann Whitney U = 74,470, *p* < 0.001).

Like the observed data, the distributions of the R^2^ values for all three permutation tests deviated from normality with a rightward skew (biological process Shapiro-Wilk = 0.67, *p* < 0.001, skewness = 2.63; molecular function Shapiro-Wilk = 0.63, *p* < 0.001, skewness = 3.19; cellular component Shapiro-Wilk = 0.62, *p* < 0.001, skewness = 2.76). As expected, the permutation tests revealed strong negative correlations between R^2^ values and category sizes (biological process Spearman’s ρ = -0.48, *p* < 0.001; molecular function Spearman’s ρ = -0.41, *p* < 0.001; cellular component Spearman’s ρ = -0.55, *p* < 0.001), indicating that the inclusion of more randomly associated transcripts and proteins has a negative impact on the predictive relationship of transcript and protein expression within a category.

### Correlation with category size

We were interested as to whether GO category size had an affect on the overall predictive nature of transcripts and proteins within a given annotation of our observed data. We found Spearman’s correlation coefficients between the categorical R^2^ value and the number of genes per GO category in our dataset. The overall correlation between R^2^ value and number of genes was negative for biological process, molecular function, and cellular component (Table 2), however this relationship was only significant for cellular compartment. The strongly negative relationship between R^2^ value and category size in cellular compartment may be due to an increase in diversity in functional processes represented within a category as the categorical size increases. The same effect was observed under the annotation of molecular function when categories were limited to those with more than 20 transcript/protein pairs. By definition, the GO annotation of “biological process” must have more than one distinct step [13] and therefore, the annotation contains genes with a greater diversity compared to the molecular function annotation regardless of the size of the category. This observation may account for the fact that no correlation is observed between R^2^ value and category size within the annotation of biological functions.

**Table 2:** Spearman rank correlation coefficients (ρ) between the transcript/protein categorical R^2^ values and possible sources of variation

		Biological Process	Molecular Function	Cellular Compartment
Category Size	All categories	−0.08	−0.05	−0.25**
	Categories with < 20 transcript/protein pairs	−0.05	−0.05	−0.26
	Categories with ≥ 20 transcript/protein pairs	0.03	−0.22*	−0.19
Abundance	Mean gene expression	−0.09	−0.17	0.10
	Mean protein expression	−0.07	0.15	0.04
Gene Length	All categories	0.05	−0.03	0.11
Synthesis/degradation	Transcription rate	0.17*	−0.31**	−0.29*
	Translation rate	0.13	0.29**	−0.30**
	mRNA half-life	−0.10	−0.36**	0.26*
	Protein half-life	0.12	0.05	−0.24

*p value of < 0.05

**p value of < 0.01

### Correlation with molecular abundance

Because low molecular expression may affect the accuracy of abundance estimates, we examined the correlation between average transcript and protein abundances and the R^2^ values for individual GO categories. We found no significant correlations between mean gene or protein expression and categorical R^2^ value, indicating molecular abundance has no bearing on the predictability of the transcript/protein relationship (Table 2).

### Correlation with gene length

Spearman correlation coefficients were found between gene length and R^2^ value. Biological process, molecular function, and cellular component categories did not produce significant relationships between these measures (Table 2).

### Production and degradation rates

Published mRNA and protein molecular half-lives and transcription and translation rates for mouse fibroblasts [8] allowed us to assess whether our observed R^2^ values were due to known differences in the molecular stability of mammalian cells. We observed strong correlations between R^2^ values and mean transcription rates, translation rates, and mRNA half-lives, respectively for categories of molecular function and cellular compartment. The same trend was not observed for biological processes, likely due to the diversity of functions included in the category as previously mentioned. The correlation between R^2^ value and transcription rate is strongly negative for both molecular function and cellular compartment, indicating that transcripts with high rates of synthesis do not have strong predictive value for downstream protein abundance.

Multiple regression analyses were performed to determine how R^2^ values were related to four variables: rates of transcript and protein synthesis and their respective degradation rates. Table 3 summarizes the results. In the multiple regressions associated with all three GO annotations, translation rate is observed to have a positive weight on R^2^ value when controlling for other variables. However, this relationship is not significant for any GO annotation. For both molecular function and cellular compartment, mRNA half-life has a larger positive weight than translation rate and is significant for both annotations.

**Table 3:** Results of multiple regression analyses of R^2^ value (dependent variable) against transcription and translation rates and mRNA and protein half-lives (independent variables)

	Estimate	Std. error	t-value	*p*
**Biological process**				
Intercept	4.23E-02	8.07E-02	0.52	0.60
Transcription rate	1.09E-02	4.06E-03	2.69	<0.01
Translation rate	8.86E-05	5.11E-05	1.73	0.08
mRNA half-life	-4.32E-03	5.60E-03	−0.77	0.44
Protein half-life	5.61E-04	1.97E-04	2.84	<0.01
R^2 ^= 0.08, F(4, 207) = 4.315, *p*-value = 0.002				
**Molecular function**				
Intercept	3.41E-01	1.02E-01	3.35	<0.01
Transcription rate	-8.63E-03	5.74E-03	−1.5	0.14
Translation rate	4.12E-05	2.89E-05	1.43	0.16
mRNA half-life	-1.38E-02	6.71E-03	−2.06	<0.05
Protein half-life	-2.58E-04	1.97E-04	−1.31	0.19
R^2^ = 0.13, F(4, 87) = 3.125, *p*-value = 0.02				
**Cellular compartment**				
Intercept	-1.89E-01	1.05E-01	-1.80	0.08
Transcription rate	-2.99E-02	8.26E-03	−3.62	<0.01
Translation rate	8.71E-05	6.15E-05	1.42	0.16
mRNA half-life	3.63E-02	6.71E-03	5.40	<0.01
Protein half-life	-8.21E-04	2.79E-04	−2.94	<0.01
R^2^ = 0.40, F(4, 63) = 10.41, *p*-value < 0.001				

Data in bold are the subcategories

### Interspecific and interregional comparisons

We previously reported the expression levels of transcript/protein pairs that are expressed in both humans and chimpanzees [6]. We used this previously published dataset of homologous transcript/protein pairs to determine whether the predictive relationship of transcripts and proteins is similar between the two species and in two different regions of the brain. Using well-represented GO categories (≥10 transcript/protein pairs) in the ACC, we found 215 annotations representing biological processes, 100 for molecular functions, and 78 for cellular components. For CN, 195, 90, and 69 annotations were found for biological processes, molecular functions, and cellular components, respectively. The confidence interval for each OLS slope was compared between humans and chimpanzees to determine if the relationship between average transcript and protein expression differed between species. We found that none of the categorical regression lines were different between species, and the ranked correlation coefficients between humans and chimpanzees were similar (biological process in ACC: Spearman’s ρ = 0.79, *p* < 0.001; CN: ρ = 0.61, *p* < 0.001; molecular function in ACC: ρ = 0.82, *p* < 0.001; CN: ρ = 0.71, *p* < 0.001; cellular component in ACC: *ρ* = 0.77, *p* < 0.001; CN: *ρ* = 0.60, *p* < 0.001). The predictive relationships between transcripts and proteins were fairly similar in both ACC and CN (biological process: ρ = 0.43, *p* < 0.001; molecular function: ρ = 0.43, *p* < 0.001; cellular component: ρ = 0.75, *p* < 0.001).

We found the absolute value of the change in rank order of the R^2^ values of GO categories between humans and chimpanzees and between regions of the brain (Additional file 1). These scores represent differing relationships between transcripts and proteins and higher values would suggest different regulatory measures acting on molecular expression [7, 14]. It is noteworthy that “cell death” and “nervous system development” are among those categories with the greatest change in R^2^ rank order between humans and chimpanzees. We note that categories such as “synapse” and “integral to plasma membrane” and those listed in Table 1 as having particularly high R^2^ values, display concordant R^2^ values across species and regions of the brain.

## Discussion

In an earlier manuscript, we reported a very low correlation between transcript and protein expression in two brain regions, ACC and CN, of both humans and chimpanzees [6]. Coefficients of determination (R^2^) were roughly 0.03 for both species and brain regions, indicating that transcript expression predicts 3% protein expression. The current study extends those initial findings by asking whether grouping transcript/protein pairs by similar attributes produces better predictive outcomes. Although we found a higher overall R^2^ value in the current study (0.07), this result must be due to the greater number of transcript/protein pairs included in the dataset. Previously, other authors have theorized that transcripts/proteins that contribute to a cell’s structure may offer a higher predictive value than those that are functionally modulated [7]. Similarly, neuronal compartmentalization may impose region-specific rates of translation in disparate areas of the cell [14]. Both of these hypotheses would favor higher predictive values across specific transcript/protein pairs compared to others as categorized by GO annotations.

We report a large degree of diversity in R^2^ values when transcripts/proteins are categorized by their function or location in the cell. We find several trends in assessing the R^2^ values of transcript and protein abundance across GO categories. First, perhaps by nature of the diverse functions and locations of the molecules contained within the categories, the annotation of biological process contains a large range of predictive values that cannot be explained by category size, molecular abundance, or molecular stability. This result may be the result of the large diversity of functions represented within each category of biological process. Under any annotation, the expression levels of transcripts grouped by GO category are capable of accounting for a maximum of 66% of the variation observed in mean categorical protein abundance. Most categories are not better or are not significantly different from random in their predictive values.

We explored possible sources of variability in R^2^ value by examining its correlation with category size, average transcript and protein expression, and gene length. We had predicted that hydrolases and other enzymes, which are typically short in length, may be particularly poor predictors of protein abundance due to their relatively fast RNA degradation [15, 16]. It is well understood that it is biologically beneficial for molecules with such function to have carefully regulated half-lives [8, 17]. Moreover, short proteins are notoriously problematic to quantify [18], making it difficult to assess whether this finding is due to technological limitations or true biological differences. We report no correlation with regard to average expression levels or gene length at the level of GO category, but these factors may contribute to variation at the level of the individual transcript/protein pair. However, the number of transcript/protein pairs within a GO category likely has an affect on the R^2^ value as evidence by the differing correlations that are apparent when GO annotations of varying sizes are considered. Specifically, the inclusion of more transcript/protein pairs may have a negative effect on the overall predictive nature of the GO category since larger categories are by their very nature less specific than small categories (ie. between 10 and 20 transcript/protein pairs). Due to the negative association between R^2^ value and category size among the largest groupings of transcripts/proteins by function and location, it may be concluded that smaller, more specific categories of transcripts may be the most predictive of their associated proteins.

The discordance between transcript and protein expression levels may be caused by differential rates of transcription and translation and/or in vivo and postmortem degradation rates of transcripts and proteins [8, 19–22]. While the stability of transcripts and proteins vary according to their functional characteristics [8, 23], the rate of translation has been found to be the most important factor in predicting protein expression [24]. At the categorical level, translation rate weighs only slightly positively on the predictive value of transcript to protein abundance. Because mean molecular synthesis and degradation rates influence the R^2^ value but not in a consistent manner across GO annotations. it is likely that when considering transcripts and proteins by GO categories these features (synthesis and degradation rates) that may otherwise help to explain molecular abundance do not have sufficient resolution. Other attributes, such as sequence features, may account for differences in predictive value [25], which are not possible to account for at the categorical level.

Despite the fact that the predictive values of many categories of transcripts and proteins are no better than random, we wish to emphasize that several categories have very high R^2^ values considering the complex dynamics of transcription rates and molecular degradation. We report that transcripts coding for protein kinases, phosphatases, and membrane-associated proteins, especially those that participate in metabolic oxidoreductase activity or the transport of ions, are among the transcripts that are most predictive of their downstream protein expression levels. It is noteworthy that many of these categories of proteins are critical for aerobic metabolism. In short, molecules supporting these specific processes may have a better correlation between their transcripts and protein expression levels than other categories.

Comparing our results to that of other studies suggests differences in the predictive value of genes across tissues. Guo and colleagues [7] found the predictive value of mRNA to protein expression in human monocytes, which were chosen for their relative homogeneity across cell types. The authors found transcript expression was poorly predictive of protein expression (R^2^ = 0.09), a result similar to our own. However, they report the highest correspondence between transcript/protein pairs in the extracellular region, whereas we found that those molecules that were intrinsic to the cellular membrane displayed the strongest correlation in expression. The extracellular categories in our dataset (“extracellular region” and “extracellular space”) are among the weaker transcript/protein correlations in the brain. Comparing the data from Guo and colleagues [7] with the current study suggests that there is a strong tissue-specific component to the relationship between transcripts and proteins. Furthermore, future work may reveal that the vast heterogeneity in neuronal transcript expression [24, 26] may also affect cellular-specific protein expression and may have profound implications for neuronal function.

We report our findings from fresh, frozen human brain tissue with PMIs of less than 8 h. It is not known how much stronger the relationship would be in fresh brain tissue. Rather than affecting all transcripts equally, postmortem degradation appears to target different classes of transcripts at varying rates [15]. Specifically, longer coding regions and 3’ UTRs correlate with more rapid degradation than the rest of the transcriptome [15, 23, 27, 28]. The most severe postmortem degradation occurs after 8 h and would not be a factor in our study [15]. It is not known how postmortem degradation affects proteins of different functional classes.

Although the current work only considers adult tissue, it is important to comment on the possibility of the relationship between transcripts and proteins changing throughout the lifespan. A recent study in prefrontal cortex of humans and Rhesus macaques found that the decoupling of transcript and protein expression increases with age and may suggest an accretion of age-dependent post-translational regulation in primate brain [12]. Like the current study, concordance was found in transcript and protein expression levels within categories enriched for nucleotide and ATP binding. In the pathways that displayed an age-related discordance in transcript and protein levels, Wei et al. [12] found that regulatory and signaling functions were enriched for mTOR signaling and metabolic functions. The authors suggest that the increasing discordance between transcript and protein expression as the lifespan progresses may be the result of mRNA binding proteins or other regulatory factors and contribute to aging and perhaps Alzheimer’s disease.

In general, our results were similar between species (humans and chimpanzees) and regions of the brain. It is likely that the predictive relationships, particularly in the categories with the highest R^2^ values, outlined here would hold true for other brain tissue in primates and perhaps mammals as a whole. We focused our attention on results from ACC in this study, but we note that our findings in CN were very similar, suggesting that the ability to predict protein expression levels from transcript expression probably do not change across different regions of the brain. Additionally, it is worth noting that the results of this study are category-specific and that the correlation of individual transcript/protein pairs contained within a category can vary.

## Conclusions

In the ACC and CN of the human and chimpanzee brain, we observe that the predictive nature of proteins can range from no predictive value whatsoever to fairly high. We find that transcripts that code for proteins that are integral to the membrane and support protein kinase and oxidoreductase activity are more predictive of protein expression than the vast majority of other categories. We conclude that it is important to consider the predictive nature of transcript/protein pairs when determining the functional implications of gene expression studies. It may be practical to consider transcript and protein expression as two separate aspects of molecular phenotype, each with its own contribution to biological function. In the future, the challenge for molecular expression studies will be to integrate transcript and protein biology into a single unified message of tissue and cell function.

## Methods

This study used transcriptomic and proteomic expression data that are available through our previously published work [6]. Briefly, frozen human brain samples (aged 34 to 51 years) were obtained from the National Institute of Child Health and Human Development Brain and Tissue Bank for Developmental Disorders at the University of Maryland (Baltimore, MD) and were free from neurological disorders. Frozen brain samples from adult common chimpanzees, *Pan troglodytes* (aged 23 to 35 years), were obtained from the National Chimpanzee Brain Resource (Washington, DC). The chimpanzees had been cared for according to Federal and Institutional Animal Care and Use guidelines and died of natural causes. All brain tissue was collected with a postmortem interval of less than 8 h and stored at -80 ° C.

Brain tissue was sampled from the ACC and CN from adult humans (*n* = 3) and chimpanzees (*n* = 3). Each sample was divided into two pieces: one for RNA-sequencing and one for quantitative proteomics. Libraries were constructed from poly-A-enriched RNA of 30 million 50-bp sequences. Orthologous gene models were constructed for each species, and sequences were mapped to species-specific genomes (hg19 and panTro3) [29, 30]. For the majority of the analyses, a dataset from human ACC was used which contained 815 transcript/protein pairs. However, to assess the variation observed between species and brain regions the expression levels of 522 transcript/protein pairs from human and chimpanzee ACC and 499 from human and chimpanzee CN were analyzed. Both transcriptomic and proteomic data were normalized in edgeR [31].

To compare R^2^ values between regions of the brain and between species, we performed Spearman rank correlations of the categories in each of the three GO annotations. In order to assess which categories represented the largest change in rank order, we found the absolute value of the difference in rank order position. These values were then scaled by dividing by the number of categories in the annotation and multiplying by 100.

In the current study, we assigned each transcript/protein pair to their GO categories for the annotations of biological process, molecular function, and cellular component. Categories contained a minimum of 10 transcript/protein pairs. We used the species mean log-transformed expression data to perform a series of linear regressions for each category. We performed OLS linear regressions rather than using the reduced major axis (RMA) method. Although RMA regressions attempt to diminish variance along x- and y-axes [32], some authors have reported that RMA can decreases variation along the x-axis that may be biologically meaningful [33, 34]. OLS only accounts for error along the y-axis [35]. We focus our report on the coefficient of determination, R^2^ values, produced from OLS regressions of each GO category individually. All statistics were performed in R (version 3.1.3) [36], and the linear regressions were performed in the SMATR package (version 3.4).

To determine if the R^2^ values of the GO categories were better than a random sampling of transcripts and proteins, we performed permutation tests in which random transcripts and proteins were classified into categories to mimic our observed data. The category sizes were sampled and replaced from the actual sizes of our observed categories for biological process, molecular function, and cellular component to ensure that the range of possible category sizes represented our dataset. The resampling occurred over 1000 iterations.

We were interested in determining whether category size, abundance levels, or gene length had an affect on the R^2^ levels produced by GO categories. We found Spearman correlation coefficients between category size (number of transcript/protein pairs within our dataset) and R^2^ value across GO annotations of biological process, molecular function, and cellular component. Next, we found mean abundance levels of transcripts and proteins and examined whether a correlation existed between these values and the R^2^. Finally, we found gene lengths by searching the RefSeq annotations for the latest human genome build, hg38, from the University of California Santa Cruz Table Browser [37]. Average gene length per GO category was compared to R^2^ value to determine if length affected transcript/protein predictive value.

We combined our transcript and protein expression data with the molecular stability measures reported by Schwanhäusser and colleagues [8]. Specifically, the authors had found transcription (molecules/[cell*h]) and translation (molecules/[mRNA*h]) rates in addition to mRNA and protein half-life time (h). The merged dataset contained molecular abundance and stability measures from 471 transcript/protein pairs. Consequently, fewer GO categories contained 10 or more transcript/protein pairs (biological process: 212 categories; molecular function: 92; cellular compartment: 68). Spearman rank correlation coefficients were found between the synthesis and degradation rates and mean categorical R^2^ value. A multiple regression was performed for each gene annotation using the R^2^ value as the dependent and the synthesis and degradation rates as independent variables.

## Additional file

Additional file 1: Complete list gene and protein expression in human ACC and the results of the OLS regressions. The file also contains the rank order changes of categories when R^2^ values are compared between species or regions of the brain. (XLSX 9357 kb)

## Abbreviations

ACC: Anterior cingulate cortex
CN: Caudate nucleus
GO: Gene ontology
LC/MS/MS: Liquid chromatography with tandem mass spectrometry
OLS: Ordinary least squares
PMI: Postmortem interval
